# Formal Verification of Heuristic Autonomous Intersection Management Using Statistical Model Checking

**DOI:** 10.3390/s20164506

**Published:** 2020-08-12

**Authors:** Aaditya Prakash Chouhan, Gourinath Banda

**Affiliations:** Discipline of Computer Science and Engineering, Indian Institute of Technology Indore, Madhya Pradesh 453552, India; gourinath@iiti.ac.in

**Keywords:** autonomous vehicles, collision avoidance, heuristic algorithm, intersection management, model checking, uppaal-SMC

## Abstract

Autonomous vehicles are gaining popularity throughout the world among researchers and consumers. However, their popularity has not yet reached the level where it is widely accepted as a fully developed technology as a large portion of the consumer base feels skeptical about it. Proving the correctness of this technology will help in establishing faith in it. That is easier said than done because of the fact that the formal verification techniques has not attained the level of development and application that it is ought to. In this work, we present Statistical Model Checking (SMC) as a possible solution for verifying the safety of autonomous systems and algorithms. We apply it on Heuristic Autonomous Intersection Management (HAIM) algorithm. The presented verification routine can be adopted for other conflict point based autonomous intersection management algorithms as well. Along with verifying the HAIM, we also demonstrate the modeling and verification applied at each stage of development to verify the inherent behavior of the algorithm. The HAIM scheme is formally modeled using a variant of the language of Timed Automata. The model consists of automata that encode the behavior of vehicles, intersection manager (IM) and collision checkers. To verify the complete nature of the heuristic and ensure correct modeling of the system, we model it in layers and verify each layer separately for their expected behavior. Along with that, we perform implementation verification and error injection testing to ensure faithful modeling of the system. Results show with high confidence the freedom from collisions of the intersection controlled by the HAIM algorithm.

## 1. Introduction

Autonomous technology is making its presence felt more and more in human life. More things are getting automated than ever. Automation not only reduces human efforts but also reduces the losses that are associated with human error and results in a system that is more safe and efficient. Autonomous vehicles are rising in popularity and they are projected to transform the traffic we see today into an intelligent group of vehicles that can communicate and cooperate with each other either using mutual negotiations or by following instructions of a central coordinator. Every vehicle in such a scenario will behave in a manner that will collectively result in optimum performance of the traffic scenario under consideration. Using this technology vehicles will be able to not only pass through any scenario in the most efficient way rather will also be able to plan ahead for coming scenarios by making favourable organizations. For instance, Reference [1] proposes a solution for intersection management of a traffic consisting of autonomous vehicles only and Reference [2] gives a cooperative algorithm for arranging vehicles in lane according to their destination direction at the intersection. Scenarios can change and with the scenario, the communication architecture, controlling policy and so forth, may change to suit the requirements. Thus, we will have a system that is much more intelligent and cooperative than present-day systems.

### 1.1. Challenges With Proving Safety of an Autonomous Vehicle Technology

For every technology, no matter how efficient or time saving it is, it has to satisfy the most important property and that property is safety. Safety is of the utmost importance when the technology is directly used by humans. That is the reason before deploying any technology for human use, it is extensively tested and verified for safety. In fact, the U.S. Department of Transportation (DoT), in its latest guideline issue [3] for the autonomous vehicle development, has put great emphasis on verification, validation, and compliance of safety standards such as ISO 26262, IEC 61508, and so forth. To prove the safety of their technology, various autonomous vehicle manufacturers use their technology to drive for a large amount of time in test drives and later use this driving time of the vehicle as a measure of the safeness of their technology. This is known as testing. Although testing is an important step in any product development life cycle, it also has its limitations. In case of autonomous vehicle technology, the extent of testing that is required to guarantee with the given confidence, the safety of operation is impractical. The study presented in Reference [4] says that to prove with 95 percent confidence that self-driving fleet has a 20 percent lower fatality rate than that for human driven, it would require 100 vehicles to drive around 24/7 for around 225 years! This calls for a technique in the development cycle that can prove the safety requirements in a reasonable amount of time unlike testing. Formal verification is one such technique.

### 1.2. The Choice of Formal Verification Technique Used

Formal verification is a systematic approach that uses mathematical reasoning to verify that the specification (requirement) is preserved in the implementation (system model). Broadly there are two classes of formal verification methods which are—(i) Property-oriented verification and (ii) Model-oriented verification. In property oriented approach, the system is modeled using a set of properties that a system satisfies; that is, the system is represented using a set of mathematical equations. The required property from this system, which is also a mathematical equation should be a logical consequence of the equations that the system satisfies. Proving this logical consequence is the basis of verifying a system in property oriented approach. This procedure involves heavy use of natural deduction and proof methods using propositional and predicate logic. Due to this reason, the property oriented approach requires expert level knowledge to obtain the proof.

The model oriented approach on the other hand abstracts mathematical equations and uses the concept of states. The system is represented as a transition system, which is a tuple containing the possible set of states, transitions between them, and the set of properties each state satisfies. Verification in model oriented approach involves exploring the given transition system model for checking the satisfiability of the required properties on states. Algorithms available for doing this are automatic and do not need human intervention or guidance to obtain results. Added with the benefit of more intuitive development of system model using graphical editor, tools present for model oriented verification offer a better choice for systems that can be represented using their underlying formal language.

Due to these differences, model-oriented and property-oriented methods have different domains of applicability. The property-oriented method is more appropriate when we do not know what the system looks like and the best way to describe them is by the means of axioms. On the other hand, the model-oriented method is more appropriate when we do know what the model looks like and we can describe them rather precisely [5]. In our case, model-oriented approach is more appropriate as we are not working with general properties, mathematical axioms, rather with we are completely aware of the system. In addition to that, the model-oriented tools present provide some features that can realize systems that are non-trivial otherwise. For instance, we have used dynamic instantiation of a vehicle template for non-deterministic arrival of vehicles, just as real world traffic.

### 1.3. The Choice of Formalism and Tool Used

Verification is a crucial part of any hardware or software system development life cycle. It is aimed towards finding errors in the design as early in the development life cycle as possible. The process of formal verification starts with the representation of the system in a formal language that is suitable for the verification technique used. The choice of formalism to model the system depends most importantly on the expressiveness that will be required to faithfully model the key dynamics of the system. For instance, a reactive system with finite states without a notion of time can be modeled using Labeled Finite State Automata (LFSA). On the other hand a time-critical system cannot be modeled using the same LFSA. They will need a formalism that can either model time or temporal ordering among the states of the system. Timed Automata (TA) and Timed Petri-Nets (TPN) are two such formalisms that have been used to model time-critical systems. Though such formalisms can indeed model timing characteristics of time critical systems, they are not panacea. In fact, real-time cyber-physical systems that involve complex dynamics and stochastic behavior are not expressible by these formalisms. Also, the model checking of such systems is undecidable and one thing that can be done is to approximate them with the available formalisms [6]. Alternatively, this problem can be solved by incorporating the formalism that can model the stochastic and non-linear dynamical behavior of the system and then exploiting the technique of Statistical Model Checking (SMC) [7]. The main idea behind SMC is to make an executable model of the system under consideration and perform a finite number of simulations. Results of these simulations are monitored and are used by the statistical techniques such as sequential hypothesis and Monte-Carlo simulations to find whether the system satisfies the required property with some given degree of confidence. One limitation of SMC is that it provides results not on the basis of exhaustive exploration, rather, a bounded number of simulations. Due to this fact, SMC is a considered as a compromise between testing and the classical model checking. Though SMC is not as powerful as the classical model checking, it is still equivalent to running an exponential number of simulations [8].

Though TPN is capable of modeling real-time and time-sensitive systems, tools present for model checking TPN models are not sophisticated enough to support statistical model checking [9]. Timed Automata, on the other hand, has tools such as Uppaal and Prism that are sophisticated, well maintained and also allow statistical analysis of the model. Apart from these, other modeling formalisms are Promela (of the SPIN model checker), CSP (of FDR model checker), TLA+ (of TLC model checker), Event-B, UML, Z, and so forth. A survey of existing tools for formal verification is given in Reference [10]. Out of all these model checkers, we have used the Uppaal model checker because of the following advantages it offers.

1. It works with models developed in Timed Automata (and their extensions) formalism.
2. It supports statistical model checking in Uppaal-SMC extension.
3. It supports the dynamic instantiation of templates
4. It supports graphical modeling which is an intuitive and easy way of modeling.
5. It offers high-level data structures and functions.
6. It is available for academic use without any cost, it is well maintained and has a big active community of users.

Out of these, features 2, 3, 4 and 5 are the ones that have been exploited in this work and their presence as a combination is the main reason for using this particular model checker.

In the presented work, we perform formal verification of the Heuristic Autonomous Intersection Management (HAIM) algorithm, which is proposed by us in Reference [1], using the Uppaal Model Checker, particularly its SMC variant. Verifying the HAIM algorithm for “No Collision” property will involve modeling traffic injection, vehicle behavior, Intersection Manager (IM), and collision detection procedure. As formal verification is aimed towards developing the system right, we perform verification at every stage of implementation of the HAIM algorithm. As we shall see, in the HAIM algorithm, we resolve conflicts in 4 stages where every following stage tries to resolve conflicts unresolved in the earlier stage. Verification is done after the implementation of each of these stages. This will guide the implementation of the HAIM algorithm and will also let us verify the claimed behaviors of each of these stages. After vehicles are scheduled by the HAIM algorithm, vehicles will travel with the assigned velocity and collision detectors will then check for collisions at every step of the simulation. Verification engine analyses these models for several runs against specified properties to check for their compliance. Furthermore, we perform implementation verification by checking the satisfiability of some invariant conditions on the execution of the model and error injection to perform sanity checking of the model. In other words, we propose and demonstrate how to exploit the advantages of SMC in formal verification and alongside verifying sane modeling of the system.

The rest of the paper is organized as follows: In Section 2, a literature survey is presented. Here we will first refer to some of the foundational articles, then we discuss some existing works that present formal verification of autonomous systems. Section 3 gives an introduction to the techniques used in this paper. Section 4 contains the HAIM algorithm which will be subjected to formal verification. Here we will discuss briefly the algorithmic steps of HAIM. In Section 5, we present the Uppaal model of the HAIM algorithm and the Collision Checker and explain the working of each constituent automaton. Properties verified and the verification results are presented in Section 6. In Section 7 implementation verification of the model is performed. Here we first define the invariant conditions over the executions of the model and then check their satisfiability. Later in the section, we perform error injection testing of the model. In Section 8, we draw inferences from results and discuss. In Section 9, we present the conclusion.

## 2. Related Work

In this section, we give a survey of literature related to the work reported in this paper. We will look at the literature related to model checking, followed by its applications in verifying autonomous systems. Then we will discuss the statistical model checking and the literature associated with it.

### 2.1. Formal Verification of Autonomous Systems

To create conceptual understanding on formal verification, readers can refer to [11,12]. These articles present basics of formal verification, discuss their applications and tools used. A comprehensive study on current state-of-the-art for formal modeling, specification and verification of autonomous systems is discussed in [13]. In Reference [14], the authors display the procedure that we have discussed earlier for verification of any autonomous system. They perform formal verification of the high level decision making software components of an autonomous vehicle called “rational agents” which are presented as being intelligent instead of reactive, and has ability to assess the situation to make the best decision. For instance, which obstacle to collide with to keep the damages to a minimum in case a collision is unavoidable. Rational agents are modeled using Gwendolen agent modeling language, and AJPF (Agent Java Path Finder) is used for formal verification of specifications written in Linear Temporal Logic. With the advancements in the autonomous vehicle technology the need for an appropriate verification technique is becoming evident. As we emphasize on application of appropriate tools and the development of strategies for formal verification, the work presented in Reference [15] has somewhat same objectives. This paper is an attempt in the direction of analysing the available options with respect to the choice of formalism and level of formality. They perform a case study over the formal verification of the Lateral State Manager module of an autonomous vehicle using three different verification approaches namely Supervisory Control Theory, Model Checking and Deductive Verification. The goal is not to compare rather differentiating based on the objective of technique and studying how multiple formalisms can help to deal with challenges in developing autonomous vehicle technology.

In the literature, the works closest to our work include Reference [16], where the authors approach the safety verification of an intersection using the KeYmaera theorem prover. They verify the safety property of the two most basic building blocks of any intersection scenario namely T-intersection/merging and two-lane intersection. The properties verified corresponding to these two cases in this work translate to (i) If the vehicle and stoplight start in a controllable state then, the vehicle will never enter the intersection while the light is red and (ii) If the stoplight and the two cars start in a controllable state, no car will enter the intersection while its light is red, respectively.

Our work differs from this work in two respects, which are (i) We present verification of the autonomous intersection management algorithm as applied on a four-way intersection with 3 lanes in either direction and (ii) We use model-theoretic verification because the model of the system involves much realistic situations such as dynamic and non-deterministic instantiation of vehicles, custom data structure and layered nature of the heuristic which makes model checking the preferred choice because of its ability to perform precise modeling of such system which would otherwise be complex and error-prone in a theorem prover such as KeYmaera. In Reference [17], authors introduce a spatial logic called Multi-Lane Spatial Logic (MLSL) and an abstract model of the multi-lane motorway based on the local view of the cars. Using the MLSL, properties needed for safety proof can be formulated and later used as guards and invariants in the design of abstract lane-change controllers. This work has been extended in References [18,19] and also in Reference [20] to model and verify traffic scenario such as intersection, turns, crossroads and T-intersections. This work is similar to the work presented with respect to the safety proof approach. The key idea behind the safety proof in both is to prove that vehicle occupy and reserve disjoint spaces. However, the difference lies in the fact that in the mentioned work, the occupancy is determined using the view of each individual car whereas, a discrete grid model is used in the presented work and this grid is updated centrally by the Intersection Manager. The discrete position of a vehicle is determined in terms of cells of grid occupied by it at any moment. Also, since vehicles travel only in their respective lanes, this grid is one dimensional (array) and each route has a corresponding occupancy array in the lane and the intersection region. The authors of Reference [21] have discussed algorithm that was used by them in DARPA urban grand challenge for collision checks in maneuvers for lane-change, overtaking, intersection crossing, and so forth. They used a point mass representation of traffic participants along with passage-time to model the length of the vehicle. Inspired from this, we, in our implementation represent vehicles as lines and model vehicle width in collision detection using extended occupancy on both ends.

### 2.2. Statistical Model Checking

Statistical Model Checking (SMC) is the extension of model checking for stochastic systems where the quantitative properties of the system are expressed in terms of measure of executions of the system satisfying certain temporal properties. The key idea behind SMC is to observe a fixed number of executions of the simulatable model of the system by certain monitoring procedure and deduce whether the system satisfies the desired temporal property or not. The results of SMC are generally associated with a bound of making the error and this is the trade off that has to be done to gain the advantage in terms of memory and time requirements [22]. SMC only requires the system to be simulatable [23] thus, increasing the class of systems that it can be applied on.

We are going to discuss next some previous works that have used statistical model checking to verify quantitative measure of the satisfaction of the required properties by any autonomous vehicle system.

Reference [24] targets the problem of formal verification of autonomous systems with a case study on the traffic sign recognition in autonomous vehicles. They define the architecture of the system in EAST-ADL which is a domain specific architectural language. This model is included with functional and non-functional properties such as time and energy constraints. To include the stochastic nature, the probabilistic extension of EAST-ADL constraints is defined and its semantics are translated to Uppaal-SMC for formal verification. In Reference [25], authors propose a verification architecture for automated cyber-physical systems. They perform two case studies corresponding to perception system and decision making system. Their main idea is to formulate some Key Performing Index (KPI) in a temporal language and use them to guide the verification using Statistical model checking. In an another work [26], the authors present verification of the functioning of controllers present in an autonomous vehicle to prevent collision in a traffic jam situation. There are two types of controllers considered; first one is only responsible for following the front vehicle without collision and the other controller has responsibility of safe changing of lane. The controllers are modeled using C++ codes and the driveway is modeled as stochastic high-level Petrinets.

As we can see in the literature survey presented above, the focus is kept primarily on one or more sub-modules of autonomous vehicles that are responsible for any dynamic driving task of the vehicle such as lane following, lane change, and so forth. This means that the current literature on the application of statistical model checking in Intelligent Transport Systems (ITS) domain has primarily been vehicle-centric. To the best of our knowledge there has not been any work in the literature that deals with the statistical verification of any traffic management algorithm such as a intersection management while considering the dynamic nature of the problem. Along with introducing the application of SMC to ITS algorithms such as intersection, in the presented work, verification done at different stages of development of the algorithm for internal verification and artificial error injection testing done to verify correct modeling of the system demonstrate a better approach towards developing a correct system.

Following are the key contributions of this paper:

- We present a novel approach to formally verify an autonomous intersection management algorithm using statistical model checking
- We demonstrate how a statistical model checker can be used in combination with the stage-wise implementation of the system to verify the correctness of implementation at every stage. This also verifies the behavior of each layer of the heuristic.
- We demonstrate how a formal model can be verified for faithful modeling using invariant satisfiability and error injection.
- We present a simplified method for representing car position and detecting collision in the lane and the intersection area. We also use the concept of extended occupancy to model the geometry of vehicles in collision detection in this simplified model.

## 3. Background Description

### 3.1. Autonomous Intersection Management

In Autonomous intersection management (AIM), the task is to schedule vehicles incoming to an intersection such that no two vehicles are present at the same space at the same time. Vehicles, which are all autonomous and can communicate with other vehicles or with a central controller using wireless communication, express their trip details in order to obtain a safe passage through the intersection. AIM is different from traffic light control in the sense that in AIM microscopic control of vehicles is generally practiced. AIM is proven to be an NP-Hard problem in Reference [27], where the authors reduce this problem to an instance of dividing a vertex set into a minimum number of independent sets. A survey of techniques for autonomous intersection management is given in Reference [28]. As the verification of any system is only as good as the model of the system, modeling of the algorithm along with the rest of the system is the most crucial aspect of verification of an AIM algorithm.

In the presented work, vehicles are treated as dynamic objects that is, they are created as instances of a dynamic template. So, for each vehicle entering the system, a separate parallel automata is created for which the model checker constructs a product automata for verification which is a computationally intensive step. This makes formal verification of an AIM algorithm non-trivial. The extent of verification (in terms of simulation time, traffic density) that we can do is also limited by this factor. We next discuss how formal modeling of the system is done and how the desired properties are specified. Uppaal SMC takes in a Stochastic Timed Automata (STA) model of the system. STA is a variant of Timed Automata hence we next give a brief introduction to Timed Automata and Stochastic Timed Automata.

### 3.2. Timed Automaton

Timed Automaton (TA) [29] is a finite state automaton that is extended with real-valued clocks. These clock values can be compared to integers to form guards for enabling or disabling transitions. TA models the temporal behavior of real-time systems. Formal definition of timed automaton is given below.

A timed automaton is a tuple TA=(L,C,Σ,E,I,AP,Guard) where
*L*: is a finite set of the locations, $l_{0}$ is an element of *L* that denotes the initial location.
*C*: is a finite set of the clocks,
Σ: is finite set called *alphabet* or *actions* of TA,
*E*: ⊆L×β(*C*) $\times \Sigma \times 2^{C}\times L$ is the set of edges,
I:L→β(*C*) assigns invariants to locations, and
AP: is a finite set of atomic propositions.
Guard:E→β(C) assigns guards to edges.

β(*C*) is the set of boolean clock constraints involving clocks from *C*. We shall write $l\overset{g,a,r}{\to }l^{\prime }$ when {$l,g,a,r,l^{\prime }$} ∈E, where $l,g,a,r,l^{\prime }$ are variables that range over L,Guard,Σ,C,andL respectively.

**Figure 1:**
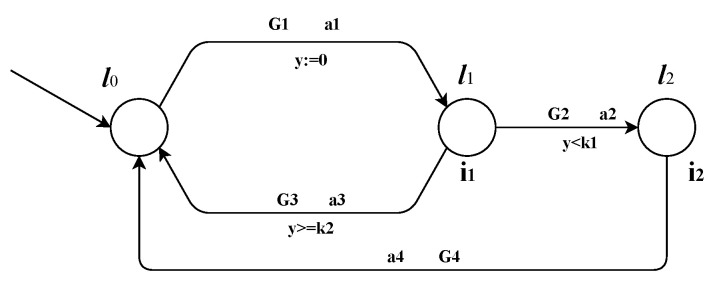
Timed automata.

Figure 1 shows an example timed automata. In this, the transition $l_{0}\to l_{1}$ occurs only when $G_{1}$ is satisfied and when it happens, action $a_{1}$ is taken and clock *y* is reset. Similarly, for the transition $l_{1}\to l_{2}$, $G_{2}$ specifies the enabling condition, $a_{2}$ is the action and condition on *y* gives the clock constraint and so on. $i_{1}$ and $i_{2}$ give invariants at the locations $l_{1}$ and $l_{2}$. Invariants are conditions that should always hold at that location. In the context of Figure 1, following are the details.

*L* = {$l_{0},l_{1},l_{2}$}
$l_{0}$ = $l_{0}$
*E* = {$\left(l_{0}\to l_{1}\right),\left(l_{1}\to l_{0}\right),\left(l_{1}\to l_{2}\right),\left(l_{2}\to l_{0}\right)$}
Σ = {$a_{1},a_{2},a_{3},a_{4}$}
*I* = {$i_{1},i_{2}$}
*C* = {*y*}

We will be using the stochastic extension of TA known as Stochastic Timed Automata (STA). STA equips TA with stochastic behavior in form of probabilistic distribution over edges and delays. Without going into the mathematical formalisms, we define STA as follows.

$$STA=(L,C,\Sigma ,E,I,AP,Guards,{\left(\mu_{q},p_{q}\right)}_{q\in L\times R_{+}^{X}})$$

where

- (L,C,Σ,E,I,AP,Guards) is a Timed Automata.
- $\mu_{q}$: Defined in terms of *q*, a state in the transition system of TA, given by q=(l,v) where *l* is a location in TA and *v* is the clock valuation, $\mu_{q}$ is a probability distribution over $R_{+}$ and it governs delay in location *q*.
- $p_{q}$: similar to $\mu_{q}$, $p_{q}$ is defined in terms of *q* and is a probability distribution over the set of edges that are enabled in *q*.

In Uppaal SMC, the delay in location is determined using a normal distribution along its valid range of time interval set by its invariant and guards on the associated edges and the choice of selection of edge is also determined using the relative probabilistic weights given to edges in the model. In Formal verification, formal model of the system is evaluated by the verification engine for the specified requirements given in a property specification language. The Uppaal model checker supports a simplified form of Computational Tree Logic (CTL) language for property specification. We next present a brief introduction to CTL

### 3.3. Computation Tree Logic

CTL defines how states of a system can evolve over time. CTL formulas consist of state operators and path operators along with logical operators. State operators express about the states of the system whereas path operators express about the paths into the future that the system can follow. The language of a well formed formula in CTL is generated using the following grammar.

ϕ::=⊤∣⊥∣p∣(¬ϕ)∣(ϕ∨ϕ)∣(ϕ∧ϕ)∣(ϕ⇒ϕ)∣(ϕ⇔ϕ)∣AXϕ∣EXϕ∣AFϕ∣EFϕ∣AGϕ∣EGϕ∣A[ϕUϕ]∣E[ϕUϕ]∣A[ϕRϕ]∣E[ϕRϕ].

All these operators take the current state as the reference for their meaning. Their meanings are given below:

- Path operators:
  - *A*: Along all paths
  - *E*: There exists at least one path
- State operators:
  - *X*: Next
  - *F*: Finally
  - *G*: Globally
  - *U*: Until
  - *R*: Release

*p* represents an atomic proposition which is the most elementary CTL expression. Let us define p1 and p2 as two atomic propositions characterizing two different events $e_{1}$ and $e_{2}$ then, the following expression will be an example of a well defined CTL formula

$$\mathrm{AG}\neg (p_{1}\wedge p_{2}).$$

This formula is to be read as “along all paths, globally that is, on all states, p1andp2 does not hold simultaneously”.

### 3.4. Uppaal Model Checker

Uppaal [30] is a verification tool suite appropriate for verification of systems that can be modeled as a collection of non-deterministic processes with finite control structure and real-valued clocks communicating through channels or shared variables. Uppaal consists of three main parts—a description language, a simulator, and a model checker. The system to be verified is modeled as a group of Timed automata. The description language is used to define variables, function definitions, and implement the inherent logic. A timed automaton consists of various locations and these locations are connected by using edges. The edges are annotated with selections, guards, updates, and synchronizations. The purpose of these annotations is explained below.

**selection:** To non-deterministically bind a given identifier to a value in a given range.
**update:** Expression that is evaluated when the edge is traversed. In Uppaal, we can use functions in update expression.
**guard:** Expression that has to evaluate to true for the edge to be traversed.
**synchronization:** Processes communicate and synchronize using channels. This annotation may work as a publisher or a receiver on the edge.

Locations in Uppaal are labeled with invariants. Invariants are expressions that should always evaluate to true for the time the system is in that particular location. Locations in Uppaal can be made Urgent or Committed. These are the locations in which time is not allowed to pass that is, they freeze time. Furthermore, if a process is in a committed location, then the next transition must involve an edge from one of the committed locations.

Uppaal requirement specification supports five types of properties given in Table 1. In this table, *p* and *q* are state properties specified with atomic proposition; *A* and *E* are path quantifiers that stand for Always and Existential respectively and [] and <> are state quantifiers that stand for ’for all states’ and ’for some state’ respectively.

## 4. Heuristic Autonomous Intersection Management (HAIM)

### 4.1. Intersection Model

**Figure 2:**
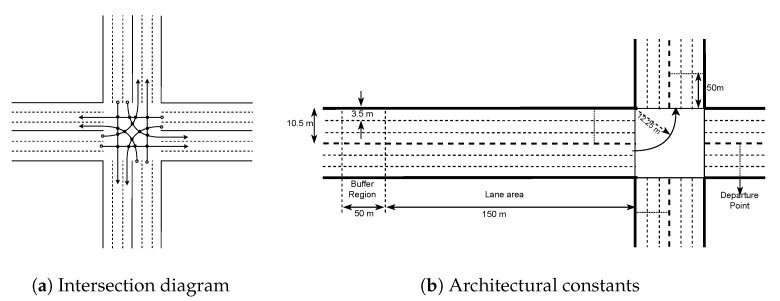
Intersection model and architectural values.

The intersection under study consists of roads in four directions. Each road has two-way traffic; one in the incoming direction and the other in the outgoing direction. Each direction has three lanes. Intersection model and values of the architectural constants are shown in Figure 2a,b.

To make the traffic more organized, some ground rules are set which are mentioned below:

1. Traffic is consisting of autonomous vehicles only.
2. Vehicles travel in the lane corresponding to their destination direction only. For example, left-turning vehicles will travel in the left lane.
3. Vehicles follow a fixed trajectory inside the intersection while turning.
4. Communication is assumed to be flawless that is, there is no packet loss and zero transmission delay.

The lane and trajectory restrictions on vehicles reduce the complexity of the system to scheduling vehicles at some fixed number of intersecting points of lane-to-lane connections here named as Conflict Points (CP). As shown in Figure 2a, there will be a total of 16 CPs and each lane to lane connection has four CPs, excepting the right turning lanes, which have none. A buffer region is kept before the lane approach area. Vehicles are required to send a message packet containing their trip details along with their velocity to the IM. IM in return will calculate the velocity with which vehicles have to travel after they leave the buffer region. IM considers the vehicle journey as starting from the end of the buffer region or the start of the approach region. For this reason, the velocity with which the vehicle will leave the buffer region is sent as the initial velocity in the message packet sent to the IM. Vehicles must receive the final velocity ($V_{final}$) by the time they are in the buffer region. As soon as vehicles leave the buffer region, they will start transiting to the velocity assigned by the IM and travel with that velocity thereafter.

To calculate V_final, IM, first of all calculates the safe lane velocity ($V_{lane}$ ). It will be the initial point for calculating velocities in the next three stages of the heuristic. $V_{lane}$ is calculated by making the departure time of the vehicle greater than the previous vehicle in that lane. We now define a function named *findVelocity()* that serves this purpose. The *findVelocity()* function returns the velocity that will make the vehicle with initial velocity v_e, cover the *target_distance* and reach at the target point at a time not less than the *target_time*. The pseudo-code for the *findVelocity()* function is given in Algorithm 1. To calculate $V_{lane}$, the departure time of the previous vehicle in lane plus the safety gap is passed as the argument *target_time*, the distance of the depart-point from the start line is passed as the argument *target_distance* and the vehicle identification number is passed as the argument v_id. We need to pass one more argument to the *findVelocity()* function and that is the maximum velocity the vehicle can have. For calculating $V_{lane}$, velocity limit in the scenario is passed in place of this argument.
**Algorithm 1:** The *findVelocity()* function
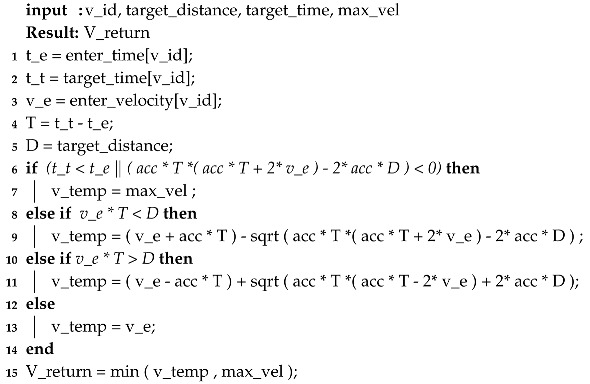


To get $V_{lane}$, the following function call is made.

$$V_{lane}=findVelocity\left(v\_id,depart\_point\_distance,last\_vehicle\_depart\_time,V\_max\right).$$

The maximum possible value of velocity is assigned in the case when it is impossible for the vehicle to reach the target point at target time with the given acceleration. This condition is checked by the expression

(acc∗T∗(acc∗T+2∗v_e)−2∗acc∗D)<0

this expression simplifies to

$$D>v\_e*T+\frac{acc*T*T}{2}.$$

This equation points to the condition in which the target distance is greater than what the vehicle can travel with the initial velocity of v_e and an acceleration of *acc* in the given time. In such a case, V_max is made the $V_{lane}$. The next two conditions inside else-ifs correspond to those cases where the vehicle can reach the target distance at the exact target time. These two conditions evaluate whether the vehicle need to accelerate or decelerate respectively from the entering velocity v_e. V_max is the maximum velocity with which a vehicle can travel and its value (17 m/s) is taken from Reference [1]; the safety gap between two reservations is kept to be 500 ms.

**Figure 3:**
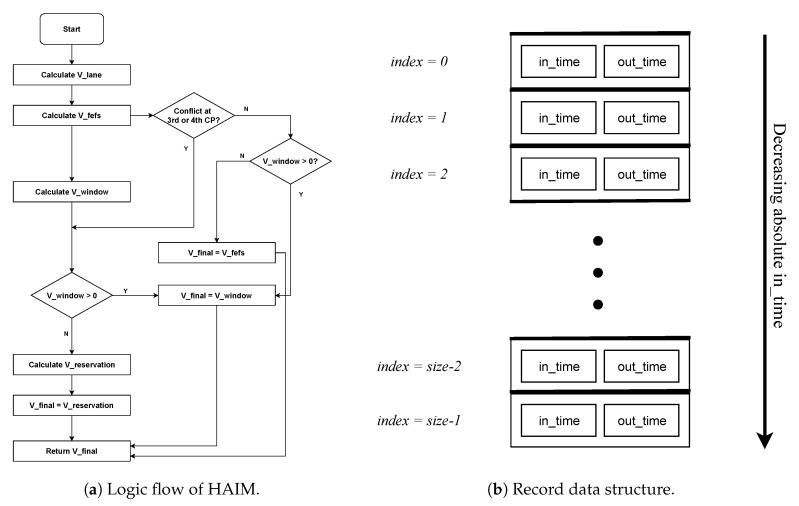
Heuristic Autonomous Intersection Management (HAIM) logic diagram and Record data structure.

After resolving lane conflicts, we move on to resolving intersection conflicts. This is done using a three-layered heuristic. These three layers correspond to: (i) First Enter First Serve (FEFS) scheme, (ii) Window scheme, and (iii) Reservation scheme. These three layers use $V_{lane}$ as their initial point and return three velocities (not necessarily different). Out of these three velocities, one of the velocity is chosen as $V_{final}$ by using the logic shown in the decision flow diagram of Figure 3a. These three layers of heuristic are explained in detail in Reference [1]; here we give their short description.

### 4.2. FEFS Scheme

In the First Enter First Serve (FEFS) scheme, vehicles are assigned reservations in the order of their arrival that is, a vehicle that entered before will always have an earlier reservation. To resolve conflict, FEFS scheme finds a velocity that will result in crossing times at the first two conflict points in the vehicle’s path such that they are greater than the previous reservations at those CPs. In other words, the FEFS scheme makes a reservation only at the top of the reservation record that is, after all the existing reservations. Any velocity that results in a reservation after the reservation at the top of the reservation record of a CP is called a satisfying velocity for that CP.

To obtain $V_{fefs}$, we use the same procedure that we used to obtain $V_{lane}$ except that here we apply it twice for each of the first two conflict points and the target time for each of these procedures will be the depart time at the top of the record of the respective conflict point plus the safety gap. The maximum value of $V_{fefs}$ for any vehicle is limited by its $V_{lane}$; hence, instead of assigning $v_{max}$, we assign the lane velocity in the first conditional check. $V_{fefs}$ will be the minimum of the velocities corresponding to the first two conflict points. The reason only the first two CPs are considered in the FEFS scheme, as discussed in Reference [1] is that only a small fraction of vehicles have a conflict on later two conflict points. Thus, finding satisfying velocity for each CP will result in unnecessary computational overhead. Pseudo-code for finding $V_{fefs}$ is given below.

v_cp1 = findVelocity (v_id , target_distance_cp1 , target_time_cp1 , V_lane [ v_id ] )
v_cp2 = findVelocity (v_id , target_distance_cp2 , target_time_cp2 , V_lane [ v_id ] )
V_fefs = min (v_cp1 , v_cp2 , V_lane [ v_id ])

v_cp1 and v_cp2 are the satisfying velocities for conflict points 1 and 2 respectively. As the velocity in FEFS scheme is limited by $V_{lane}$, $V_{lane}$ for that vehicle is passed in place of the max_vel argument.

### 4.3. Window SCHEME

In contrast to the FEFS scheme, the Window scheme can make reservations at any position in the reservation record. As the name suggests, in this scheme, a window is searched in between the previous reservations. $V_{lane}$ is taken as the initial point. The velocity at which a window is obtained at all the conflict points will be returned as the $V_{window}$. As reservation can be done at any position in reservation record, the order in which vehicles arrive is not preserved in the Window scheme. This will enable any fast-moving vehicle that entered later to pass the intersection before a slow-moving vehicle that entered earlier. As discussed in Reference [1], this breaks delay piles generated by the FEFS scheme at higher traffic rates. Please note that the Window scheme can only make a reservation if a window is available at all conflict points. In case no window is available, this scheme will not be able to return any velocity. Also, if the Window scheme returns a velocity, then it is will always be conflict-free which is in contrast with the FEFS scheme in which the velocity returned is not guaranteed to be conflict-free. Pseudo code for finding $V_{window}$ is shown below in Algorithm 2.

The function $isWindowAvailable(cp_{i},vel,d\_cp_{i})$ searches entire record of the given conflict point ($cp_{i}$) which is at a distance $d\_cp_{i}$ from the start line to search whether the travel time corresponding to the given velocity (vel) falls inside a window or not. It returns true when a window is present for the given velocity in the reservation record of all four conflict points; otherwise, it returns false.
**Algorithm 2:** The algorithm to find $V_{window}$
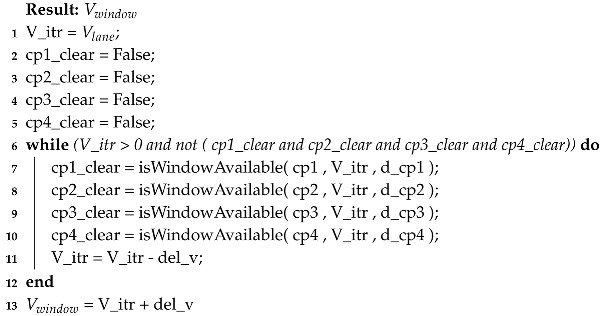


### 4.4. Reservation SCHEME

Reservation is the traditional reservation scheme in which all four conflict points are resolved before making the reservation. The velocity returned by the Reservation scheme is guaranteed to be conflict-free. However, the FEFS scheme and Window scheme are put ahead of the Reservation scheme because both these schemes return a velocity which is greater than the one returned by the Reservation scheme. Also, having a Window scheme prevents the piling of delays in the system. In the Reservation scheme, conflicts are resolved for third and fourth conflict points. The minimum of satisfying velocities of third and fourth conflict points and $V_{fefs}$ will give the reservation velocity, $V_{reservation}$. The procedure is same as that for finding $V_{fefs}$ except that instead of cp1 and cp2, cp3 and cp4 are resolved for conflicts.

v_cp3 = findVelocity (v_id , target_distance_cp3 , target_time_cp3 , V_lane [ v_id ])
v_cp4 = findVelocity (v_id , target_distance_cp4 , target_time_cp4 , V_lane [ v_id ])
  V_reservation = min(v_cp3 , v_cp4 , V_fefs [ v_id ])

v_cp3 and v_cp4 are the satisfying velocities for conflict points 3 and 4 respectively.

We mentioned in our previous paper [1] that each of these layers serves a specific purpose. In essence, As V_reservation is calculated by resolving conflicts at all the four conflict points, it is the velocity that guarantees no collision of vehicles, however, it is not the maximum velocity that a vehicle can have in various cases. Here $V_{fefs}$ and $V_{window}$ comes into the picture. $V_{fefs}$ only considers the first two conflict points instead of all four. As pointed out in Reference [1], at low traffic rates, only a few vehicles have conflict at the last two conflict points. Thus $V_{fefs}$ is obtained in less computation than $V_{reservation}$. On the other hand, Window scheme breaks delay pile generated by the FEFS scheme at high traffic and also attempts to further reduce the delay caused in the FEFS scheme. Detailed discussion on behavior of these layers is given in Reference [1]. In the next section, we model the complete heuristic, vehicle behavior, its movement through lane and intersection, lane collision detector and intersection collision detector using the Uppaal-SMC model checker and check its correctness. The logic flow of HAIM is as shown in the flowchart in Figure 3a.

The data structure used for making and storing reservations is shown in Figure 3b. We call this data structure as *record*. Each element of the record will have two fields corresponding to the *in-time* of the vehicle at that CP and the *out-time* of the vehicle at that CP. The size of the record can be roughly set as the estimate of the number of vehicles in the scenario as the vehicle under consideration can only have a conflict with vehicles already in the scenario, not the ones that have already left or the ones still to enter. We take its size to be 10 that is, at every CP, record of previous 10 reservations will be kept. The elements of the record are always kept in decreasing order of in-time that is, top of the record (having index 0) will have the largest absolute in-time. With every new reservation, one element will be added to the record. We can add an element at any position in the record. However, since FEFS and reservation schemes only grant reservations after all the existing reservations, they can only make reservations at the top of the record. Whereas, the window scheme can insert an element at any position in the record. Always an insertion in the record is followed by shifting of all the following elements by one position and the last element gets deleted.

## 5. Statistical Model Checking With Uppaal-SMC

### 5.1. HAIM Modeling in Uppaal-SMC

For systems that need dynamic creation and termination of automata and/or needs as an output the estimate of a probability, the standard Uppaal model checker will not suffice. For this reason, we choose Uppaal’s Statistical Model Checker (SMC).

Uppaal-SMC [31] performs several runs of the system then uses results from statistics to get an overall estimate of the correctness of the system with respect to a property. Uppaal-SMC is different from the traditional Uppaal in the way that it allows us to specify the probability distribution that drives the timed behavior and also that the SMC engine offers the output of any query in probabilistic terms. The engine can: (i) Estimate the probability of an event, (ii) Compare the probability of an event with a value, and (iii) Compare two probabilities without computing them individually.

In Uppaal-SMC, to include the probabilistic nature, the query language is enhanced with the weighted version of the Metric Interval Temporal Logic (MITL) [32] which is defined by the following grammar.

$$\phi ::=p|\neg \phi |\phi_{1}\wedge \phi_{2}|O\phi |\phi_{1}U_{\leq d}^{x}\phi_{2},$$

where *p* is an atomic proposition, *x* is a clock, *d* is a natural number, O is the next operator, U is the Until operator and the logical symbols have their usual meanings. The weighted formula $\phi_{1}U_{\leq d}^{x}\phi_{2}$ is satisfied by the run if $\phi_{2}$ is satisfied before the clock *x* exceeds *d*, and until that, $\phi_{1}$ is satisfied. Two derived temporal operators known as time constrained *eventually* (⋄), and time constrained *always* (□), are defined as follows.

$${⋄}_{x}^{\leq d}\phi =true\;U_{d}^{x}\;\phi and{□}_{x}^{\leq d}\phi =\neg \;{⋄}_{x}^{\leq d}\neg \phi .$$

The eventually operator is defined in terms of the Until operator and the always operator is defined in terms of the eventually operator. This allows us to write the following four types of queries in Uppaal-SMC.

(1) *Quantitative analysis or probability estimation:* Estimates the probability of a path expression being true.
  *For ex.*Pr[<=K]{[]ϕ}, will give probability of ϕ to be true at every state when the system is run for *K* number of steps.
(2) *Qualitative model checking or hypothesis testing:* Checks whether the probability of a property is less than or greater than the specified bound.
  *For ex.*Pr[<=K]{[]ϕ}>=P, will state whether probability of ϕ to be true at every state when the system is run for *K* number of steps is greater than *P* or not.
(3) *Probability comparison:* Compares two probabilities indirectly without estimating them.
  *For ex.*$\mathrm{Pr}\;\left[<=\;K\right]\;\{\left[\right]\;\phi_{1}\}<=\mathrm{Pr}\;\left[<=\;K\right]\;\{\left[\right]\;\phi_{2}\}$, will compare the two probabilities and return truth value of the expression.
(4) *Value estimation:* Estimates the value of an expression by running a given number of simulations.
  *For ex.*E[<=K]{Var}, will give the estimated value of the variable Var.

We next discuss the modeling of HAIM in Uppaal-SMC. We model our Intersection system using two automata, which are (i) Traffic Automaton and (ii) Master Automaton. The Master automaton is divided into three sections depending on the functionality, these three sections are:

1. Vehicle Initialization Section,
2. Controller Section, and
3. Movement and Collision Check Section

We will next discuss these automata individually.

#### 5.1.1. Traffic Automaton

**Figure 4:**
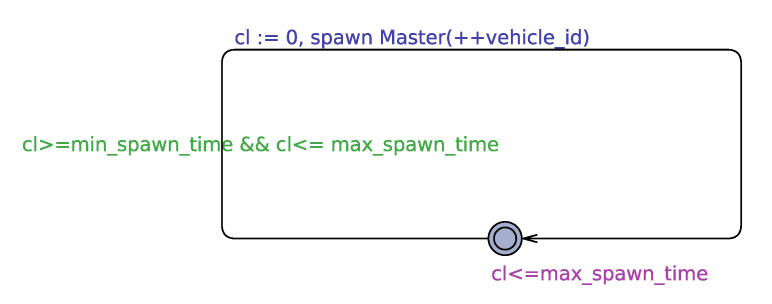
Traffic automaton.

Traffic automaton releases new vehicles into the system. The release of vehicles is done after a non-deterministic time interval given by the bound in Figure 4. The bound is fixed to generate a traffic density of a minimum of 500 vehicles per hour and a maximum of 5000 vehicles per hour. This density range is the same as that used in the HAIM paper [1]. Since real-world traffic is non-deterministic, we can say that our traffic induction method is very close to the real-world scenario. We call this release as the spawning of vehicles. With a simulation step of 0.1 s, *min_spawn_time* and *max_spawn_time* come out to be 7 (closest integer) and 72. To obtain these numbers we divide the total number of steps in one hour (which is 36000) by the number of vehicles to be spawned in one hour that is, 5000 and 500 respectively.

#### 5.1.2. Master Automaton

Master automaton is declared as a dynamic template; that means, the automaton is instantiated when a vehicle is spawned in the system. Dynamic templates are declared using the *spawn* function as shown in the Traffic automaton in Figure 4. To terminate a dynamic template, *exit* command is used Also, there can be multiple instances of a dynamic template present at one time that means multiple master templates can exist at the same time with different levels of progression within their automaton.

**Figure 5:**
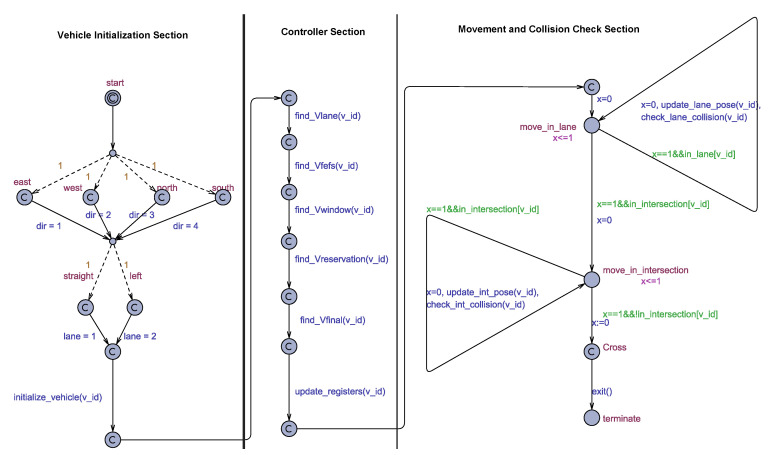
Master automaton.

The Master automaton is shown in Figure 5. It has been divided into three sections depending on their functionality for the explanation. We next discuss these three sections individually.

#### 5.1.3. Vehicle Initialization Section

The Master automaton starts in this section as the initial location is in this section. The initial location is followed by dashed edges which represent probabilistic edges with the relative weights specified as annotations. These dashed edges will randomly decide the source lane and the destination lane of the vehicle. All the weights are kept the same to get equally distributed traffic. The *initialize_vehicle()* function initializes the vehicle using the values of *dir* and *lane* variables. This function resolves the indexes of the conflict points that lie in the path of this vehicle and distances between successive conflict points are also assigned in this function. The arrival velocity of the vehicle is set randomly (less than maximum velocity).

#### 5.1.4. Controller Section

In this section, the task is to implement the HAIM pipeline. The pipeline starts with calculating the lane velocity for the vehicle. Lane velocity is then used as the starting point for calculating the rest of the three velocities namely FEFS velocity, Window velocity, and the Reservation velocity. To decide which of these three velocities will be the final velocity, we implement the logic flow given in the flowchart in Figure 3a, in the function *find_Vfinal()*. It is this section of the Master automaton in which stage-wise implementation of HAIM algorithm is done. The find_Vfinal() function is attached to the outcomes of find_Vlane(), find_Vfefs(), find_Vwindow() and find_Vreservation() respectively in the corresponding implementation stage. We discuss more about this in the next section.

#### 5.1.5. Movement and Collision Check Section

The modeling of the HAIM algorithm in the Controller section of the Master automaton uses committed states to model the assumption that all of the communication and computation has been flawless and the vehicle has received its $V_{final}$ that is, the velocity the vehicle has to attain after it leaves the buffer region. The movement of the vehicle starting from the end of the buffer region is modeled inside the Movement and Collision Check section of the Master automaton.

After leaving the buffer region, the vehicle will first travel in the lane and first of all, make a transition from the entering velocity to the assigned velocity ($V_{final}$). At each step, the position and velocity of the vehicle are updated. For the time the vehicle is travelling in the lane, the Master automaton will be in the location *Move_in_lane*. When the vehicle comes inside the intersection area, the automaton will come into the *Move_in_intersection* location. Functions *update_lane_occupancy* and *update_int_occupancy* update the position of the vehicle in the lane and the intersection occupancy vectors respectively. These positions are used by the lane and intersection collision detection routines.

To detect lane collisions, we use the discrete position of the vehicle given by the lane occupancy vector of the corresponding lane. Lane occupancy vector is an array of cells. Those cells which coincide with the position of the vehicle have a value of 1. To determine the occupied cells for a vehicle, we first find the cell corresponding to the head position of the vehicle and then set all the cells to 1 that comes under the length of the vehicle. For simplicity, we have used in the simulation, the same length (3 met.) for all the vehicles. The procedure *update_lane_occupancy()* implements this logic.

**Figure 6:**
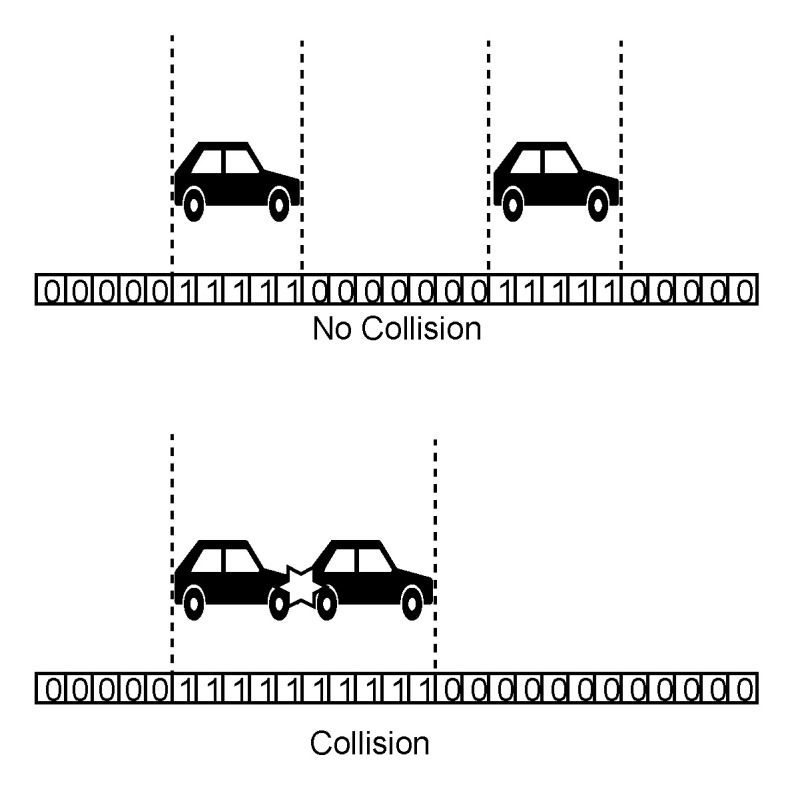
Lane collision detection.

Now, to detect a collision, we will look for the number of cells for which there is continuous occupancy. If the length corresponding to continuous occupancy of cells crosses the length of one vehicle then that would mean there is a collision between two vehicles. Illustration is shown in Figure 6. Complete overlap of occupancy of two vehicles would go undetected, however, with the limits of velocity and the dimensions of vehicles used, it would not be possible for two occupancies to be non-overlapping at one step and completely overlapping at the very next step because that would require relative distance of at least the length of the vehicle (3 m) to be covered in one times step which is greater than maximum possible value (1.7 m). The collision condition is checked for each lane occupancy vector at every simulation step. For any positive detection, a lane collision counter is incremented. The function *checkLaneCollision()* performs the above mentioned operation. The guard, invariant and update of clock variable *x* make sure that the collision is run at every step of the simulation. When the vehicle is through the intersection area, the Master template will be terminated.

**Figure 7:**
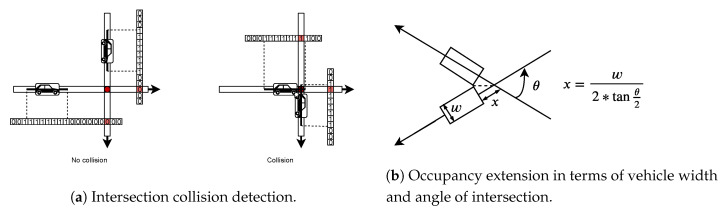
Intersection collision detection logic and occupancy extension to model vehicle width and angle of intersection

The intersection collision detection routine works in the same fashion as that of lane collision detection. Here also, the position of the vehicle inside the intersection is represented using discrete cells inside the intersection occupancy vector. As we have modeled vehicle occupancy using line segment, the width of the vehicle is not modeled. This was not a concern in the lane collision detection as vehicle always follow the order in which they entered. However, in case of collision detection in an intersection, the width has to be modeled. Along with width, we also need to model the position of vehicle in the intersection due to the angle of intersection at the conflict point. Angle of intersection is as depicted in Figure 7b. To model these two, we use extended occupancy of vehicles. This means, the line segment corresponding to the length of the vehicle is extended on both ends by a width given by the expression shown in Figure 7b, this figure also shows how this expression models the geometry of vehicle at the conflict point. The occupancy of cells of these vectors is updated at every step for each vehicle in the simulation. Like in lane collision detection, we calculate the position of the cell corresponding to the head of the vehicle and then find the trailing occupancy cells using the length of the vehicle. Implementation is done inside the function update_int_occupancy().

To detect the intersection collision, we use a different approach than in the case of lane collisions. The cells corresponding to all the conflict points are determined in all the lane to lane connections. Width of vehicles is incorporated in collision detection by extending the intersection occupancy of vehicles on both sides by a length given in terms of the width of the vehicle in Figure 7b. This way we detect the intersection collision of vehicles using their occupancy only at common conflict point. Simultaneous occupancy by two vehicles of the cell corresponding to a common conflict point will mean that there is a collision in the intersection. If that happens, an intersection collision counter is incremented. The function *checkIntCollision()* performs the above mentioned operation. The guard, invariant and update of clock variable *x* make sure that the collision check is performed at every step of the simulation. Figure 7a illustrates the working of the intersection collision detection routine.

Table 2 lists all the functions used in the Uppaal-SMC model of the HAIM algorithm.

## 6. Verification

To perform the sanity check of the modeling of the HAIM algorithm in Uppaal-SMC, we perform verification to verify the claims made about the properties of the reservation made by the three constituent layers along with verifying the safety property for the complete heuristic. To do this, we model the HAIM algorithm in the following stages.

(1) Model with only $V_{lane}$.
(2) Model with $V_{lane}$ and $V_{fefs}$.
(3) Model with $V_{lane}$, $V_{fefs}$ and $V_{window}$.
(4) The complete model with all the velocities.

At each of these stages, queries are run to get the probability (confidence) of the model in resolving lane and intersection collisions. Since we put the collision count in counters called lane_collisions and int_collisions, to determine the confidence of model in resolving collisions, we encode a query that has a literal meaning as: *What is the probability that the count of the given counter is always zero when the model is run for a bounded (given) number of steps*. In Uppaal-SMC, this query when translated for lane collision and intersection collisions both look as

$$\begin{array}{c} Pr\;[<=\;K]\;\{[]\;lane\_collisions==0\},and\\ Pr\;[<=\;K]\;\{[]\;int\_collisions==0\} \end{array}$$

These are the queries that are passed to the model checker. Here, *K* is the number of steps for which the model is run. We perform verification for values of *K* ranging from 1000 to 5000, this way we can observe the evolution of the behavior of the model at each stage of implementation. Choosing a larger value of *K* is constrained by the time the model checking tool takes to return results. For K=5000, the verifier took more than 90 days to give output for query corresponding to the complete model.

Verification results are tabulated in Table 3. This table contains verification results corresponding to five values of *K* which are 1000, 2000, 3000, 4000, and 5000. For each value of *K*, there is a verification result corresponding to the 4 implementation stages mentioned above. Verification results corresponding to no lane collision and no intersection collision are given in columns 3 and 4 of the table respectively. Columns 5 and 6 give results for error injected systems as explained in Section 7.2.

### 6.1. Model with Lane Velocity As the Final Velocity

The lane velocity is calculated by making the depart time of the current vehicle greater than that of the previous vehicle in the same lane. This velocity is the maximum velocity with which the vehicle can travel that will not result in a collision in the lane. However, it can result in a collision inside the intersection. Thus the claim made about the model with lane velocity as the final velocity is that the vehicle will not undergo any lane collision but it can result in an intersection collision. Thus the statistical model checker should return the maximum possible confidence value for no lane collision but not for no intersection collision. Verification results are given in Table 3 in rows corresponding to $V_{lane}$.

### 6.2. Model with FEFS Velocity As the Final Velocity

The FEFS velocity is calculated by resolving conflicts only for the first two conflict points. So, there is a possibility that a slow-moving vehicle that entered before the current vehicle has a reservation at the third or the fourth conflict point at a time which overlaps with the current vehicle’s schedule. Such conflicts are not resolved in the FEFS scheme so when the FEFS scheme is used alone, it may result in intersection collisions. However, since the FEFS scheme starts from the lane velocity and goes in the decrement mode to find FEFS velocity, we can say that FEFS velocity is always less than or equal to the lane velocity. Hence, there should not be any lane collisions. Also, since the FEFS scheme resolves various intersection collisions, the probability of no intersection collision is expected to be more than that in the previous stage. Verification results are given in Table 3 in rows corresponding to $V_{fefs}$.

### 6.3. Model with FEFS Velocity and Window Velocity

Window scheme performs a window search operation within the previous reservations and its main aim is to further reduce the delay after the FEFS scheme by finding a greater velocity than the FEFS velocity if possible. It is claimed in the HAIM algorithm that the Window velocity is guaranteed to return a collision-free velocity if it returns a velocity. And in case it cannot find a window, the $V_{fefs}$ will be the final velocity. So at this stage, the final velocity is given by window scheme if the $V_{window}$ exists; otherwise, the $V_{fefs}$ is assigned as the final velocity.

Since both FEFS and Window schemes return a velocity that is smaller than or equal to the Lane velocity, there should not be any lane collision caused in this stage as well. Also, as some of the conflicts unresolved by the FEFS scheme are resolved by the Window scheme, we expect a greater probability of no intersection collision than in the previous stage. Verification results are given in Table 3 in rows corresponding to $V_{fefs}\;\mathrm{and}\;V_{window}$.

### 6.4. Complete Model with FEFS, Window and Reservation Velocities

Here we have all the constituent layers of HAIM, that means, this model should result in a collision-free trip through the lane and the intersection. The logic flow shown in Figure 3a is used to decide the final velocity. Verification results are given in Table 3 in rows corresponding to $V_{fefs}$, $V_{window}$ & $V_{reservation}$.

## 7. Implementation Verification

So far, we have introduced the HAIM algorithm, modeled it in the formalism of probabilistic timed automata using the Uppaal-SMC tool and presented verification results corresponding to four different stages of implementation. All this has been presented with an underlying assumption that the modeling of the HAIM algorithm is done faithfully. In fact, the majority of works in literature have this underlying assumption. This assumption leaves a possibility of false positive. In an attempt to prove the sanity of the modeling performed, correctness verification of the modeling itself is presented in this section.

### 7.1. Invariant Satisfiability

To prove that the model of HAIM correctly captures its properties, we define invariant conditions that model should satisfy at every instant of the run. These properties are directly inherited from the HAIM algorithm itself. In other words, these properties represent invariant conditions over the execution of the algorithm and their fulfillment would mean faithful translation of the system. Following is the list of properties verified.

1. $0\leq V_{fefs}\leq V_{lane}$:
  As we have previously, $V_{fefs}$ is always less than $V_{lane}$. In fact, $V_{lane}$ is greater than $V_{window}$ and $V_{reservation}$ as well hence the next two properties follow.
2. $0\leq V_{window}\leq V_{lane}$
3. $0\leq V_{reservation}\leq V_{lane}$
4. $V_{reservation}\leq V_{fefs}$:
  As $V_{reservation}$ considers all four CP instead of the only first two and as a result $V_{reservation}$ is the velocity that is minimum in all satisfying velocities of 4 conflict points.
5. $V_{reservation}\leq V_{window}$ (if $V_{window}$ exists):
  As $V_{window}$ finds window between reservations below the top of the reservation record, $V_{window}$ is greater than $V_{reservation}$ if $V_{window}$ exists.

Properties queried in the model checker and their results are given in Table 4. Variable names can be understood from the above mentioned properties as they are presented in the same order.

**Figure 8:**
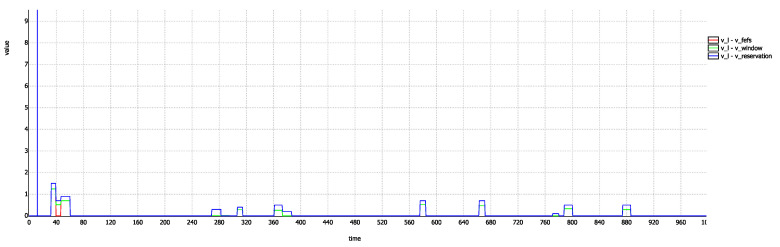
Difference of other velocities from $V_{lane}$.

Along with verifying the above specified properties using the model checker, we also perform a simulation to visualize these properties. For this purpose, we plot the difference of the $V_{fefs}$, $V_{window}$ and $V_{reservation}$ from $V_{lane}$ as shown in Figure 8. A non-negative value throughout this graph shows that $V_{lane}$ is always greater than the rest of the three velocities. Also, the relative magnitudes can be compared as specified by above properties. We can also notice one thing from this graph and it is that $V_{fefs}$ almost coincides with $V_{reservation}$ except only at a few time steps. This tells us that only at few occasions, $V_{fefs}$ is different from $V_{reservation}$. This confirms our claim that most of the conflicts are resolved at first two conflict points.

Values of the probability outputs given in Table 3 shows that vehicles actually cross the lane and intersection regions and reach their destination. However, to prove it, we insert in the Master automata, a state called *Cross* before the Terminate state. We then ask the simulator that how many templates are currently in the scenario and how many templates has reached the *Cross* state. To get the output, the following query is passed to the verifier.

simulate1[<=500]numOf(Master),sum(v:Master)(v.Cross)

**Figure 9:**
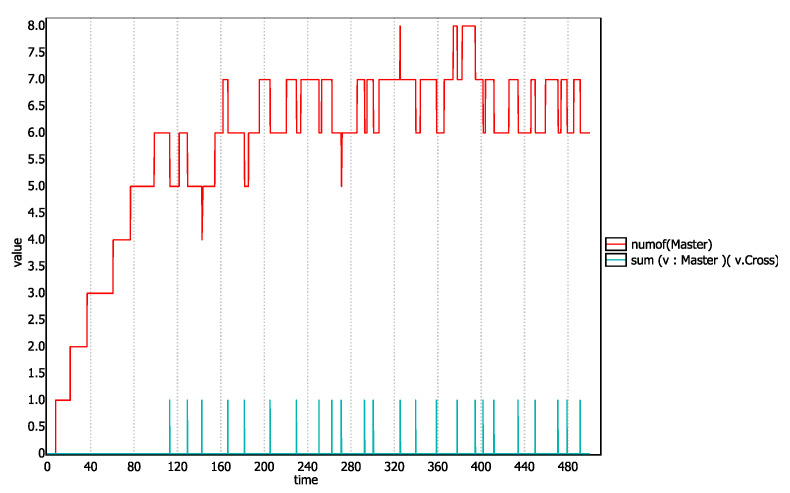
Number of active templates at any given time and templates getting terminated.

The graph that this query generates will depict the number of *Master* templates present at any given time in the simulation and also will give a spike of truth value of 1.0 every time a master template reaches the *Cross* state. The graph is shown in Figure 9. We can observe in this graph each spike of the truth value *v.Cross* corresponds to a decrease in the graph of *numOf(Master)*. This shows that *Master* templates actually traverse through the lane and intersection and get terminated.

### 7.2. Artificial Error Injection Testing

We will now deliberately inject errors into our model and see whether the verifier reflects the error in the model or not. If the error is reflected that should mean that the model has deviated from a setting that was indeed correct. One could argue that if a system is incorrect, then introducing some error will make it more incorrect and hence, the error injection result should not prove anything. However, in the context of the presented subsection, where verification results have given affirmation of the correctness of the algorithm and also algorithm’s behavior is proved to be preserved by the model as established by invariant satisfiability, error injection will indeed strengthen the confidence in overall implementation. To inject errors, we choose Vehicle and Controller (IM) as these are the two prominent entities. To inject an error in the IM, we choose the maximum satisfying velocity of all 4 CPs instead of the minimum. This way the IM will resolve conflict only at one CP whereas, on other CPs, conflicts may occur. The results obtained confirm that the error injected system is making overlapping reservations which result in collisions as evident by the low probability in the output given in the second last column of Table 3.

To model vehicle malfunctioning, we make some (every tenth) of the vehicles in the simulation disobedient. These vehicles will just travel with their initial velocity disregarding other vehicles and the intersection manager. Outputs corresponding to this error injection is given in the last column of Table 3.

The entire verification related source codes and system setup details have been uploaded on to GitHub and accessible via the link: https://github.com/aadiprakash163/HAIM_verification.

## 8. Discussion

Results given in Table 3, along with verifying the HAIM algorithm, also verify the claimed behavior of the HAIM algorithm. Starting with the first stage of implementation which corresponds to $V_{lane}$ only, we obtain maximum confidence for no lane collision and non-maximum confidence for no intersection collision, which is what we expected. As all other velocities are less than or equal to $V_{lane}$, we would expect that all of them result in maximum confidence in no lane collision. In the second stage of implementation, we use $V_{fefs}$ only. As $V_{fefs}$ resolves some of the intersection conflicts but not all, we would expect the confidence for no intersection collision at this stage to be not maximum but more than that in the case of $V_{lane}$. Similarly, as $V_{window}$ resolves some of the conflicts unresolved by $V_{fefs}$ but not all, we expect at this stage greater confidence for no intersection collision but not maximum. The final stage shall resolve all the conflicts hence, it should result in maximum confidence for no lane collision as well as no intersection collision. As we can see in Table 3, all these expectations are met in each set of experiments corresponding to different values of *K*. Though the numbers presented in Table 3 does not contain much significance when considered alone. However, the evolution of these results with an increasing number of steps and with an increasing level of implementation of the algorithm, tells us about the behavior of the stages of implementation.

The methodology adopted to represent vehicles and their occupancy inside lane and the intersection has a small memory footprint, results in very efficient collision detection, and captures essential dynamics even in the limited resources of the verification tool. The geometry of the vehicle while crossing the conflict point is considered using the angle of intersection and extended occupancy. By performing the verification of the model, we demonstrate how internal verification can be done and argue about its importance in the complete verification. Apart from satisfying invariants, we perform two error injections in the system, one in the IM and another in the vehicle. However, the first error injection in which the IM is made to work in an erroneous manner should result in more number of collisions. This is because the IM will make incorrect reservations for all the incoming vehicles. Whereas, in the second error injection, only some of the vehicles are made disobedient. Due to this reason, we expect the probability of no intersection collision in the first erroneous system to be less than the second one. Meeting of all these expectations added with the proof of termination of Master templates, gives us the evidence of faithful modeling of the overall system. In fact, the stage-wise implementation and verification has helped us a lot in surfacing errors in implementation numerous times during the development of the HAIM model.

The query we give to the verifier translates to, “*What is the probability that there is no lane/intersection collision at all when the system is simulated for K number of steps*”. Since $V_{lane}$, $V_{fefs}$ and $V_{window}$ do not guarantee the no-intersection-collision condition, the probability for no-intersection-collision for the first three stages of implementation does not show maximum confidence. Also, this probability should decrease with an increase in the number of steps. Same is the case with error injected systems as well. For a simulation with a sufficient number of steps, these values should reach the minimum confidence range of the verifier however, the choice of simulation steps is restricted by the verification time. Though the work presented considers verification for 5000 steps, it demonstrates how we can model an intersection scenario, vehicle behavior, and collision detection and verify with limited resources of a model checker.

Verification results obtained for the model corresponding to the complete model with all four heuristic-levels give absolute confidence in resolving all conflicts and giving a collision free intersection control implementation. The confidence shown by the verifier for the complete heuristic is [0.99, 1] which represents the absolute confidence by the verifier. This output shows that the actual probability of no collision in the scenario can be anything between 0.99 and 1.0, both values included. This absolute confidence is shown only when in all the runs, verifier could not find any counter example and that is the reason it includes a probability of 1.0 in its result. Had been the case that it found even a single counter-example, we would not have received this output. In SMC, the required precision can be adjusted to trade off with the number of runs verifier performs to give results which is a direct measure of the verification time the verifier takes.

## 9. Conclusions

In this paper, we investigated the open problem of verifying autonomous intersection manager. Keeping in mind the inherent stochastic nature of certain autonomous technologies, we performed a statistical model checking of the Heuristic Autonomous Intersection Management (HAIM) algorithm using Uppaal-SMC. We performed both, requirement verification by proving the safety requirements and model verification by artificial injection of errors in the model. The HAIM algorithm is modeled as consisting of three layers of heuristic. Requirement verification is performed at all these layers to demonstrate the claimed behavior of these layers. Results can be stated as

*The probability that there is no collision in the lane as well as in the intersection for 5000 steps when the HAIM algorithm is used to schedule the passing of the non-deterministic traffic through the scenario is at-least 0.99*.

In addition to that, we perform implementation verification of the HAIM using invariant satisfiability, error injection testing and verify that vehicles actually terminate. In total, in this work, we present a systematic approach of modeling an AIM algorithm, injecting traffic non-deterministically (or deterministically), simplified representation of vehicles and collisions in conflict point based scenarios, formal verification of AIM algorithms and implementation verification of the model using the Uppaal-SMC model checker. Our future work will be directed towards formally modeling the cooperative behavior of vehicles and optimization based decision making algorithms to formally verify the Cooperative Lane Sorting algorithm given in Reference [2].

## Figures and Tables

**Table 1:** Properties in Uppaal.

Name	Example
Possibly	E<>p
Invariantly	A[]p
Potentially always	E[]p
Eventually	A<>p
Leads to	p−−>q

**Table 2:** Functions used in Uppaal model.

Serial no.	Function Name	Description
1	*initialize_vehicle()*	Initialize variables such as enter_vel, cp_indices, cp_distances, etc.
2	*find_Vlane()*	Iterate to find safe lane velocity.
3	*find_Vfefs()*	Iterate to find velocity that satisfies critical CP at FEFS stage.
4	*find_Vwindow()*	Iterate to find velocity that find window between present reservations.
5	*find_Vreservation()*	Iterate to find velocity that satisfies critical CP at reservation stage.
6	*find_Vfinal()*	Implements the logic flow diagram shown in Figure 3a.
7	*update_lane_occupancy()*	Update lane position of vehicle in the lane occupancy vector.
8	*update_int_occupancy()*	Update intersection position of vehicle in the intersection occupancy vector.
9	*check_lane_collision()*	Checks in each lane occupancy vector for length of continuous occupancy.
10	*check_int_collision()*	Checks for simultaneous occupancy of CP by two vehicles.

**Table 3:** Verification results.

Steps	Velocities Used	Pr(no_lane_col)	Pr(no_int_col)	Pr(no_int_col) in Erroneous System (IM)	Pr(no_int_col) in Erroneous System (Veh)
	$V_{lane}$	[0.99, 1]	[0.772123, 0.792123]		
	$V_{fefs}$	[0.99, 1]	[0.971126, 0.991126]		
1000	$V_{fefs}$ and $V_{window}$	[0.99, 1]	[0.982451, 1]	[0.757588, 0.857588]	[0.848374, 0.948374]
	$V_{fefs}$, $V_{win.}$ & $V_{res.}$	[0.99, 1]	[0.99,1]		
	$V_{lane}$	[0.99, 1]	[0.567835, 0.587835]		
	$V_{fefs}$	[0.99, 1]	[0.950781, 0.970781]		
2000	$V_{fefs}$ and $V_{window}$	[0.99, 1]	[0.974259, 0.994259]	[0.599051, 0.699051]	[0.721003, 0.821003]
	$V_{fefs}$, $V_{win.}$ & $V_{res.}$	[0.99, 1]	[0.99, 1]		
	$V_{lane}$	[0.99, 1]	[0.411410, 0.431410]		
	$V_{fefs}$	[0.99, 1]	[0.930020, 0.950020]		
3000	$V_{fefs}$ and $V_{window}$	[0.99, 1]	[0.965653, 0.985653]	[0.424255, 0.524255]	[0.664092, 0.764092]
	$V_{fefs}$, $V_{win.}$ & $V_{res.}$	[0.99, 1]	[0.99, 1]		
	$V_{lane}$	[0.99, 1]	[0.296923, 0.316923]		
	$V_{fefs}$	[0.99, 1]	[0.912165, 0.932615]		
4000	$V_{fefs}$ and $V_{window}$	[0.99, 1]	[0.954518, 0.974518]	[0.317209, 0.417209]	[0.563821, 0.663821]
	$V_{fefs}$, $V_{win.}$ & $V_{res.}$	[0.99, 1]	[0.99, 1]		
	$V_{lane}$	[0.99, 1]	[0.213275, 0.233275]		
	$V_{fefs}$	[0.99, 1]	[0.89438, 0.914348]		
5000	$V_{fefs}$ and $V_{window}$	[0.99, 1]	[0.947572, 0.967572]	[0.263008, 0.363008]	[0.48794, 0.58794]
	$V_{fefs}$, $V_{win.}$ & $V_{res.}$	[0.99, 1]	[0.99, 1]		

**Table 4:** Properties checked for invariant satisfiability.

S.no	Property	Confidence
1	Pr([] [0, 1000] $V_{fefs}$ >= 0 && $V_{fefs}$ <$V_{lane}$)	[0.99, 1]
2	Pr([] [0, 1000] $V_{window}$ >= 0 && $V_{window}$ <$V_{lane}$)	[0.99, 1]
3	Pr([] [0, 1000] $V_{reservation}$ >= 0 && $V_{reservation}$ <$V_{lane}$)	[0.99, 1]
4	Pr([] [0, 1000] $V_{reservation}$ <= $V_{fefs}$)	[0.99, 1]
5	Pr([] [0, 1000] $V_{window}$ != 0 –>$V_{window}$ >$V_{reservation}$)	[0.99, 1]
